# Please give me a copy of my child’s raw genomic data

**DOI:** 10.1038/s41525-021-00175-y

**Published:** 2021-02-17

**Authors:** Lauren Chad, Michael J. Szego

**Affiliations:** 1 Clinical and Metabolic Genetics, The Hospital for Sick Children, Toronto, ON Canada; 2 Department of Bioethics, The Hospital for Sick Children, Toronto, ON Canada; 3 Department of Paediatrics, University of Toronto, Toronto, ON Canada; 4 Centre for Clinical Ethics, Unity Health Toronto, Toronto, ON Canada; 5 The Centre for Applied Genomics, The Hospital for Sick Children, Toronto, ON Canada; 6 Dalla Lana School of Public Health, Departments of Family and Community Medicine and Molecular Genetics, University of Toronto, Toronto, ON Canada

**Keywords:** Molecular medicine, Paediatrics

## Abstract

In this work, we explore whether raw genetic data generated during sequencing ought to be returned to a pediatric patient and/or their parents/guardians. We identify the principles used by various professional societies in their guidelines on the return of secondary findings and apply them to this new context. We conclude that since each situation is unique, decisions should be made on a case-by-case basis according to the best interests of the child.

*A child with multiple congenital anomalies and developmental delay undergoes clinical whole exome sequencing. Although no genetic etiology is found, the patient’s father requests the raw data for future re-analysis*.

This seemingly simple request may not even raise ethical alarm bells. But the request for and release of raw sequence data is uncharted territory in the genomics era. Should the clinician comply with the father’s request? Over the past few years, next-generation sequencing (NGS), which includes clinical exome and genome sequencing, have become transformative diagnostic tools in pediatrics^1–4^. These technologies can analyze all or part of an individual’s genome at once and may shorten a diagnostic odyssey. As costs fall, the use of NGS is likely to become even more widespread^5^.

NGS generates large amounts of data including the assembly, alignment, and variant calling that creates a high-quality sequence file. Raw data can be thought of as the genomic sequence of an individual before it undergoes interpretation and before a report is generated that will become part of the patient’s health record.

There has been extensive debate on the return of secondary findings in the pediatric context^6–11^. Whether *raw* sequence data should be returned, either directly to patients or parents/guardians, or to an ordering physician has not been examined with as much scrutiny. The catch, of course, is that the return of raw sequence data implies the return of *all* the data, including data a patient may have opted out of receiving or data that ought not be returned to children or their parents/guardians, based on previous recommendations^12–14^. In this commentary we explore whether raw data ought to be returned to patients or parents/guardians. We identify four principles from published guidelines on the return of secondary findings and apply them to the context of raw data. We suggest that the pediatric context is very nuanced and that decisions on whether to return raw data to patients or parents/guardians should be made on a case-by-case basis based on the best interests of the child.

## Culture of data sharing

There has been support from academics and the general public to make raw data available to participants and patients^15,16^. For example, The Global Alliance for Genomics and Health recommended providing access to individuals who request their raw genomic data^17^. This position is aligned with a recent legal and ethical analysis suggesting patients and research subjects should have access to their raw data, provided there are no compelling moral reasons to override such a request^18^. However, support is not unanimous with some authors suggesting not returning raw data or at minimum requiring certain conditions be met (e.g., resources from funders) before release is justified^19,20^.

Individual health record access rights are being strengthened under data privacy laws such as the European General Data Protection Regulation^21^. Whether this extends to a patient’s right to access their raw sequence data is still uncertain. Sequencing facilities in Europe report receiving a small number of such requests, which are handled on an ad hoc basis^21^. In North America, many clinical laboratories now have transparent consent processes to release raw data to clinicians or individuals. A recent analysis from 18 such laboratories showed that 44% allowed for raw data to be returned^22,23^. Laboratories that allow for data release are supported by The American Civil Liberties Union position that raw data should be considered personal health information that laboratories ought to return upon request^24^. In the pediatric context, several providers specified that they would return raw data to parents/guardians with their consent^22^.

In our experience at a tertiary care pediatric hospital, parents occasionally ask for their child’s raw NGS data, which is currently not part of the health record. For many parents, this is just a natural extension of the increased availability of health information accessible through online patient charts^25^, occurring within this broader movement favoring increased genetic data sharing. However, even within a data-sharing culture, there is the recognition that not all health information should be shared on these portals (e.g., tests revealing misattributed paternity)^26^.

## Return of data policies: from governing bodies to local practices

Currently, there is a lack of guidance on how to deal with requests for raw data return, particularly in a pediatric context. The closest analogs are guidelines for the return of secondary findings from The American College of Medical Genetics (ACMG), the European Society of Human Genetics (ESHG), and the Canadian College of Medical Geneticists (CCMG)^13,14,27^. While each jurisdiction may take a different approach, the guidelines are meant to help clinicians and laboratories decide what findings ought to be offered to patients.

The ACMG has the most permissive policy and recommends that, with patient consent, secondary findings in 59 medically actionable genes should be returned to patients, in addition to primary test results^13,28^. Importantly, while the majority of these conditions are adult-onset, the ACMG also recommends returning these variants when found in children as the results may have immediate implications for family members and for the child when they are older^13^.

In contrast, the ESHG and CCMG assume that, as a general rule, the harms of returning secondary findings outweigh the benefits. These organizations take a cautious approach and suggest employing a bioinformatics pipeline that minimizes the identification of secondary findings. However, they also recognize that labs may want to search for secondary findings and the CCMG provide guidance on what results to return, including variants associated with highly penetrant conditions that are medically actionable in childhood^14,27^.

## Ethical considerations of raw data requests from parents or guardians

To help clinicians who may be faced with requests for raw data release, we identified the principles underlying the guidelines for the return of secondary findings and applied them to the context of returning raw data. Before naming and describing these principles, we should acknowledge the influence of principlism in western bioethics. Principlism is an approach that employs four principles: respect for persons, beneficence, non-maleficence, and justice^29^. These principles form the backbone of influential reports such as the recommendations by the Presidential Commission for the Study of Bioethical Issues report on secondary findings, which was released the same year as the ACMG and ESHG guidelines^30^. These principles overlap with the four principles we argue are underpinning the ACMG, CCMG, and ESHG guidelines, which we apply to the context of returning raw data:

### Patient/guardian choice

Respecting patient autonomy underlies the ACMG, CCMG, and ESHG guidelines, but in different ways. For patients undergoing clinical NGS, the ACMG recommends patients of all ages be offered testing for a list of medically actionable variants but must provide consent for their return^13,31^. The ACMG also highlights the importance of parental decision-making. The CCMG takes a more cautious approach specifying that parents should receive genetic results associated with medically actionable results in childhood. Results associated with medically actionable adult-onset disorders should be only made available if requested by the parent/guardian when the information could prevent serious harm to the health of a parent or other family members^14^. The ESHG points out that providing adult-onset risk information to parents may undermine the child’s future right to decide^27^.

Patient choice is aligned with the ethical principle of autonomy. Capable patients have the authority to make medical decisions which could include requests to receive their raw genomic data. In the pediatric context, when a child lacks decision-making capacity, their parent or legal guardian makes medical decisions for them, although the child would ideally be included in the decision-making process to the extent possible. During conversations around return of results, children should be included and their views should be elicited when appropriate, to respect their emerging autonomy. If raw data is returned to parents of children without decision-making capacity, parents should be informed of their ethical obligation to inform their child that they have their raw genomic data when they are capable of making their own treatment decisions. The concerns around the potential undermining of the child’s future autonomy should also be discussed with parents who request raw data.

Both the ACMG and CCMG suggest that parental autonomy is important when making decisions about returning secondary findings given the potential health implications for the patient or other family members^13,14^. However, it is important to note that both guidelines also place restrictions on parent/guardian choice to maximize the possibility of benefit and minimize the possibility of harm. This concept is further developed in the next section.

### Clinical utility/beneficence

The ACMG specifies that variants be returned where they could provide medical benefit, in the form of prevention or treatment, for patients and their families^13^. The CCMG also recognizes that preventing serious harm to family members is a rationale for returning secondary findings; however, they place greater importance to returning variants for conditions that are medically actionable in childhood^14^. The ESHG identifies that decisions balancing respect for future autonomy with the provision of potentially life-saving information are a complicated calculus^27^. This is made more difficult by the inherent challenges in measuring and comparing the possible clinical utility of variants associated with childhood-onset disorders and those associated with adult-onset disorders in the child and adult family members. Overall, however, the CCMG, ACMG, and to a lesser extent the ESHG guidelines suggest a broader, relational notion of best interests which take into account the well-being of family members as they relate to the pediatric patient^9,11^.

There is already a recognition that pediatric genomic data sharing can benefit present and future patients, with ethical guidance emerging on how data sharing should occur^32^. While these guidelines are intended for researchers and clinicians, with raw data release, parents and children themselves could also play a role in promoting increased data sharing, provided it was done with the appropriate institutional review board oversight. Increased data sharing could help facilitate future research endeavours and help create the larger data trusts needed in this era of ‘big data’.

As our knowledge grows and interpretive software improves, re-analysis of NGS data could “solve” some initially undiagnosed cases. Parents could use available online tools or seek re-analysis through healthcare providers. In addition, while returning a child’s carrier status is contentious and not currently recommended in the pediatric context^33^, one can imagine a moment where access to such information may have some utility for that child when older or for other family members. Provided there was a possibility of benefit, one could argue that raw data ought to be returned for the potential benefit or future benefit that may come from data sharing, reanalysis, or new uses of genomic data.

### Minimizing the risk of harm

The ACMG prioritized genes deemed medically actionable in their list of secondary variants to be reported to patients^13^. Although not explicitly stated, one interpretation of ACMG’s minimal list is an attempt to reduce the risk of harm to patients. Only the variants with the best evidence of pathogenicity and an available treatment or prevention strategy are returned. The CCMG highlighted the potential emotional toll of returning secondary findings to patients who undergo NGS^14^. Both the CCMG and ESHG suggest not searching for secondary findings in the first place as a way to minimize harm^14,27^.

The risks of harm associated with releasing raw data are different in scope when compared to returning secondary findings. First, there are inherent privacy risks if a child’s raw data were shared, uploaded, or even just stored inappropriately. With the proliferation of consumer genomic databases, genomic data should be considered as identifiable information^34,35^, which can result in patient identification and misuse. If results were available to insurance companies, employers, or members of the child’s social circle, this may result in differential treatment based on their genetic background.

There is also more potential for misinterpretation of raw data. In a healthcare setting, variants likely associated with the primary indication for testing are returned; secondary variants are returned depending on parent/guardian choice. These results, which require high-quality interpretation from the lab and clinician, are typically returned by a geneticist with the support of a genetic counselor. In contrast, raw sequence data is likely returned to parents/guardians without knowing or disclosing its (potentially harmful) contents. Following raw data release, analyses may be performed by parents/guardians or other healthcare professionals, some of whom may lack the training or expertise to understand findings. Having non-experts perform genomic analyses may lead to the misinterpretation of variants as harmful. Alternatively, true findings may be discovered but without immediate access to healthcare providers to contextualize the information and plan the next steps. Both circumstances could lead to psychological harm to both the parent/guardian and patient.

Parents who obtained their child’s raw data would need to decide whether to disclose to their child that they have their raw data and account for what they did with it. This disclosure could impact the parent–child relationship since the child may feel like their parents have information about them that they do not have the right to know or have access to. While the potential for harm will be patient and family-specific, thought should be put into the risk of harm before any data is released. Parents and clinicians will need to work together to decide whether the risk of harm justifies the release of raw data, and work to minimize these risks.

### Responsibility to third-party family members

The ACMG, CCMG, and ESHG mention third-party relatives in their guidelines^13,14,27^. One of the ACMG’s justifications for returning secondary findings is to provide medically important information for the child *and the child’s family*^13^. The CCMG, while not generally supportive of returning secondary variants, states that preventing serious harm to the health of a parent or family member is one justification for their return^14^. Similarly, the ESHG recognizes that the “right not to know” is not absolute in cases where the health interests of family members are at stake^27^. Releasing raw genomic data could also have implications for family members. Individuals requesting raw data should be informed of this and encouraged to speak with family members about whether they would want to know any medically relevant findings if found.

## A case-by-case approach based on the best interests of the child

**Fig. 1:**
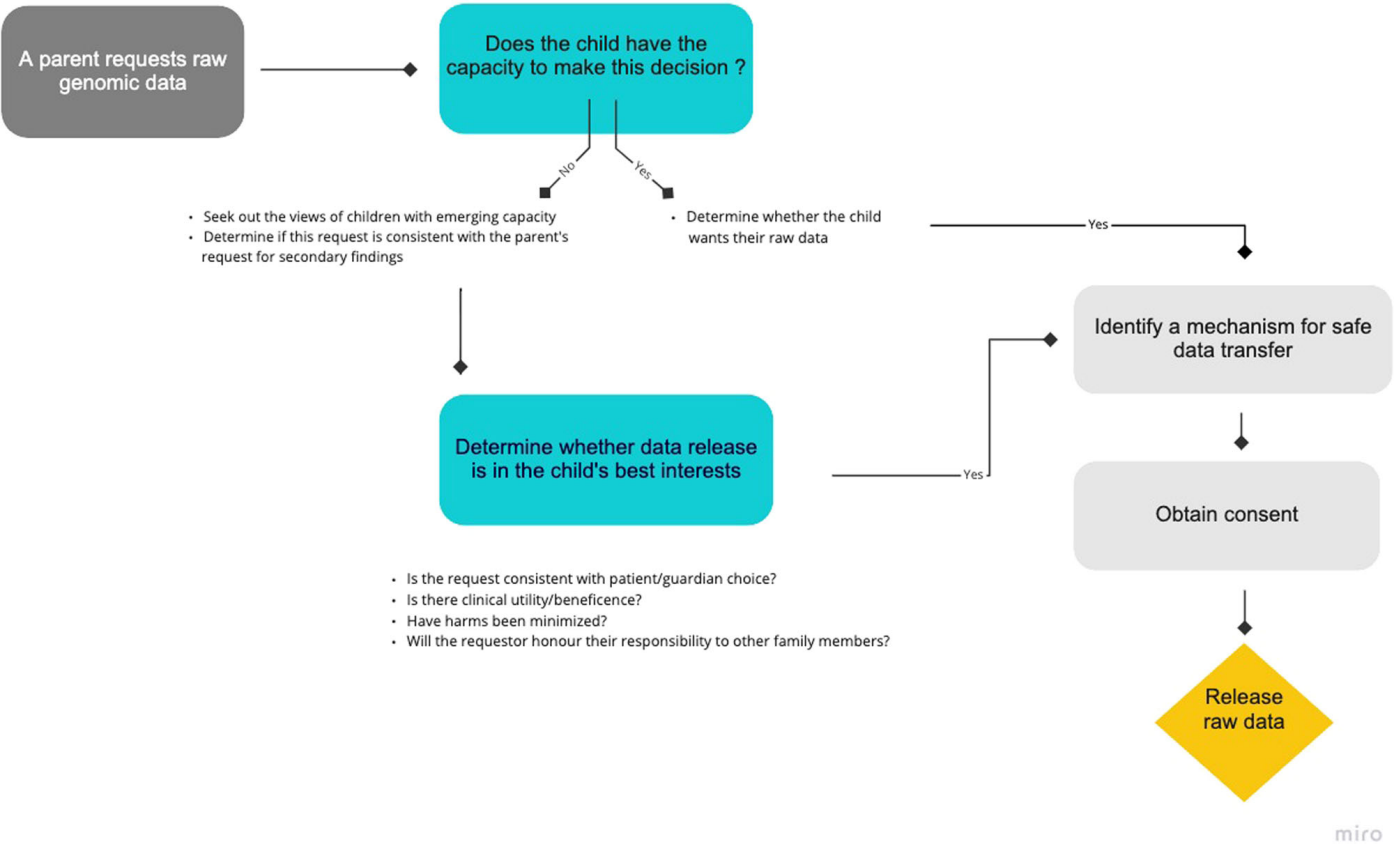
Framework and approach to raw genomic data requests. This figure guides the clinician through the various steps involved in determining whether raw data should be released. It captures important elements such as assessing the child’s capacity, considering the best interests of the child, and obtaining informed consent. This process is meant to occur in a setting that involves appropriate genetic counseling.

We have tried to describe some of the potential risks and benefits of raw data release, though we recognize that each case is unique and will require deliberation. Given all the complexities and unique circumstances of each request, we are recommending that decisions regarding the release of raw data should be made, at least at present, on a case-by-case basis with the best interests of the child at the center of these discussions. As part of the deliberation (Fig. 1), the ordering clinician would be responsible for discussing the option of raw data release with the individual who requested the data, and involving the patient when appropriate. The risks and benefits of raw data release should be explored along with strategies to minimize risks for patients and for family members. Following these discussions, the ordering clinician would determine whether the release of raw genomic data is in the best interests of the child. Best interests—a moral and legal requirement when making decisions for incapable children—require decision-makers to assess the immediate and long-term needs and select the option that maximizes the benefits and minimizes the harms to the pediatric patient^36^. Beneficence and the principle of minimizing the risk of harm overlap with this concept.

Should raw data be released, it ought to be done in a setting involving genetic counseling and it should be transferred to parents in a secure manner and in a format that is meaningful to families. One would expect that the request for raw data would come from patients/parents who also wanted secondary findings returned. If patients do not want to receive secondary findings, but would like to receive raw data, the reasons behind these seemingly inconsistent decisions should be discussed. There may be situations when parents request raw data to share with a research team, but may still wish not to know about secondary findings, highlighting there is no ‘one fits all’ approach.

## Conclusion

We identified four ethical principles underlying guidelines for the return of secondary findings, and then applied them to raw sequence data return in the pediatric setting. By identifying the principles of patient/parent preference, beneficence, minimizing the risk of harm, and responsibility to third-party family members we also allow for their application in other novel circumstances. For example, as genome-wide sequencing is becoming more accessible and available for critically ill children, a foreseeable scenario involves the parents of a deceased child requesting raw data release. Healthcare providers could take the principles identified here and apply them to this and other circumstances to make an ethically justifiable decision about raw data release. In such scenarios, there might be a need to think critically about whose best interests are at play.

Work to explore the current landscape for returning raw data amongst clinical labs is ongoing. However, whatever the current practice may be, the genetics community—both research and clinical—need to develop an anticipatory infrastructure to be able to respond to the generation of and request for raw data. Creating secure, patient accessible data repositories for genomic data is one piece of necessary infrastructure to facilitate genomic data sharing with patients. These repositories will require technical solutions and ongoing ethical and legal discussion. Since the analysis we have presented is focused on who should have access to raw data, we believe these principles will still be relevant as the necessary data-sharing infrastructure is put in place.
