# Author Correction: Please give me a copy of my child’s raw genomic data

**DOI:** 10.1038/s41525-021-00191-y

**Published:** 2021-03-23

**Authors:** Lauren Chad, Michael J. Szego

**Affiliations:** 1 Clinical and Metabolic Genetics, The Hospital for Sick Children, Toronto, ON Canada; 2 Department of Bioethics, The Hospital for Sick Children, Toronto, ON Canada; 3 Department of Paediatrics, University of Toronto, Toronto, ON Canada; 4 Centre for Clinical Ethics, Unity Health Toronto, Toronto, ON Canada; 5 The Centre for Applied Genomics, The Hospital for Sick Children, Toronto, ON Canada; 6 Dalla Lana School of Public Health, Departments of Family and Community Medicine and Molecular Genetics, University of Toronto, Toronto, ON Canada

**Keywords:** Molecular medicine, Paediatrics

Correction to: *npj Genomic Medicine* 10.1038/s41525-021-00175-y, published online 17 February 2021

The original version of this Article contained an error in the author affiliations.

Affiliation number 2 incorrectly read ‘Department of Bioethics, The Hospital for Sich Children, Toronto, ON, Canada'

This has now been corrected in both the PDF and HTML versions of the Article.

